# Biological Importance of Complex Sphingolipids and Their Structural Diversity in Budding Yeast *Saccharomyces cerevisiae*

**DOI:** 10.3390/ijms252212422

**Published:** 2024-11-19

**Authors:** Motohiro Tani

**Affiliations:** Faculty of Applied Biological Science, Gifu University, Yanagido 1-1, Gifu 501-1193, Japan; tani.motohiro.i3@f.gifu-u.ac.jp

**Keywords:** sphingolipid, complex sphingolipid, ceramide, long-chain base, budding yeast, *Saccharomyces cerevisiae*

## Abstract

Complex sphingolipids are components of eukaryotic biomembranes and are involved in various physiological functions. In addition, their synthetic intermediates and metabolites, such as ceramide, sphingoid long-chain base, and sphingoid long-chain base 1-phosphate, play important roles as signaling molecules that regulate intracellular signal transduction systems. Complex sphingolipids have a large number of structural variations, and this structural diversity is considered an important molecular basis for their various physiological functions. The budding yeast *Saccharomyces cerevisiae* has simpler structural variations in complex sphingolipids compared to mammals and is, therefore, a useful model organism for elucidating the physiological significance of this structural diversity. In this review, we focus on the structure and function of complex sphingolipids in *S. cerevisiae* and summarize the response mechanisms of *S. cerevisiae* to metabolic abnormalities in complex sphingolipids.

## 1. Introduction

Sphingolipids are eukaryotic membrane lipids with a long-chain base (LCB) as their basic structure [1]. A structure containing LCBs and fatty acids is called a ceramide (Cer), and when a hydrophilic head is added to Cer, it becomes a complex sphingolipid (in this review, sphingolipid is used as a term including LCBs, LCB 1-phosphates, Cers, and complex sphingolipids). All eukaryotes synthesize Cer and have complex sphingolipids as components of their biomembrane lipids. Sphingolipids play important roles in regulating intracellular and extracellular signal transduction systems, membrane dynamics, intracellular vesicular trafficking, and acquirement of stress tolerance [2,3,4,5,6]. Plasma membranes are composed of many types of membrane lipids, but their distribution is heterogeneous. In particular, complex sphingolipids and Cers form lipid microdomains, often referred to as lipid rafts, by functioning cooperatively with sterol molecules [4,6]. The main reason that sphingolipids can form microdomains is thought to be that, unlike glycerophospholipids, their fatty acid moieties are mainly composed of saturated fatty acids. Therefore, lipid microdomains are generally considered to be highly ordered and tight-packed structures composed of sphingolipids and sterols. Specific proteins involved in signal transduction systems and some transporters are concentrated in lipid microdomains, and thus, lipid microdomains provide a platform for these proteins to function efficiently [4,6]. On the other hand, in humans, many genetic diseases are known to cause an abnormal intracellular accumulation of specific sphingolipids due to a deficiency of lysosomal sphingolipid hydrolases and their activator proteins, which are generally called sphingolipidoses [7]. In addition, mutations in sphingolipid metabolic enzymes that localize, other than lysosomes, also cause various genetic diseases [8,9,10,11]. These facts support the notion that sphingolipids have essential roles in cellular functions.

The budding yeast *Saccharomyces cerevisiae* has several advantages in molecular biological approaches, greatly contributing to the identification of sphingolipid biosynthetic enzyme genes and the elucidation of sphingolipid metabolic regulation mechanisms. For example, the cloning of genes encoding serine palmitoyltransferase (SPT), 3-ketodihydrosphingosine reductase, and Cer synthase, which are enzymes involved in the biosynthesis pathway of LCBs and Cers common to all eukaryotes, was first reported in *S. cerevisiae* [12,13,14,15]. In addition, the regulation of SPT activity via Orm1 and Orm2, which are thought to play central roles in sphingolipid homeostasis in biological membranes, was first discovered in *S. cerevisiae* [16,17]. This mechanism has also led to the understanding of sphingolipid metabolic control via ORMDL1–3 in mammals [16,18]. Furthermore, *S. cerevisiae* has contributed to identifying enzyme genes involved in the degradation of Cers and complex sphingolipids, as well as the metabolic pathway from LCBs to acyl-CoA through phosphorylation of LCB [8,19,20,21,22,23]. On the other hand, it should be noted that the hydrophilic head group of complex sphingolipids differs greatly between species; that is, in *S. cerevisiae*, inositol phosphorylceramide (IPC) is generated by transferring inositol phosphate from phosphatidylinositol (PI) to Cer, whereas in mammals, choline phosphate is transferred from phosphatidylcholine to Cer to synthesize sphingomyelin [24]. However, it is noteworthy that mammalian sphingomyelin synthases have been first identified from the common sequence motif of IPC synthase (Aur1) homologs and the lipid phosphate phosphatase family proteins [25].

Complex sphingolipids consist of three structural units: a hydrophilic head group, LCB, and fatty acid, each of which has structural diversity, resulting in a large number of structural variations in complex sphingolipids [1]. For example, mammals are thought to have over 1000 molecular species of complex sphingolipids. This structural diversity provides an important molecular basis for the various physiological functions of complex sphingolipids. For example, lactosylCer alpha-2,3-sialyltransferase gene-knockout mice that cannot synthesize the ganglioside GM3 exhibit phenotypes such as abnormal insulin sensitivity and complete hearing loss [26,27]. However, the overall physiological significance of complex sphingolipid structural diversity remains poorly understood. However, the structural variations of complex sphingolipids in *S. cerevisiae* are much simpler than those in mammals [2,28]. Leveraging this fact, many studies have been conducted to understand the physiological significance of complex sphingolipid structural diversity. In this review, we focused on the relationship between the structure and physiological functions of complex sphingolipids based on the molecular genetics of *S. cerevisiae*. By summarizing these issues, the goal is to deepen the comprehensive understanding of why a single organism possesses a diverse range of complex sphingolipid structures. In addition, mechanisms that counteract metabolic defects in complex sphingolipids are discussed. Understanding the defense response mechanisms against abnormalities in complex sphingolipids will be important for understanding the biological significance of complex sphingolipids themselves.

## 2. Biosynthesis Pathway of Complex Sphingolipids in *S. cerevisiae*

**Figure 1:**
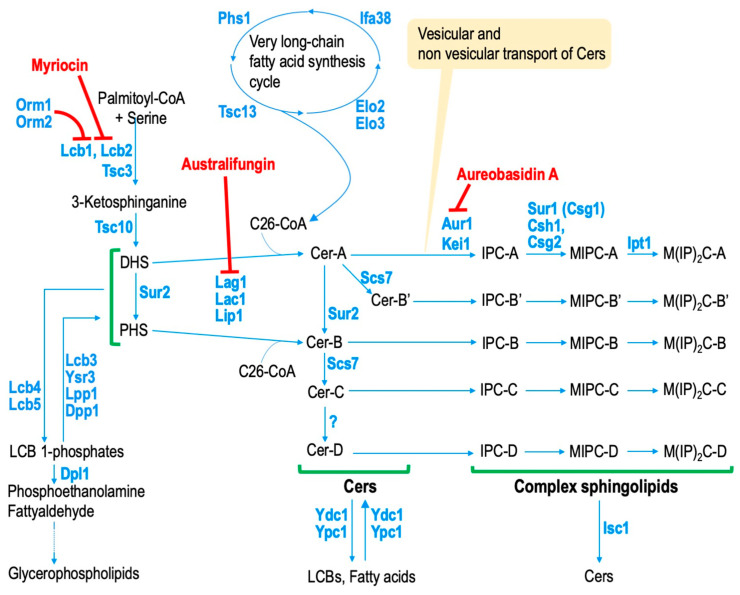
Metabolic pathway of complex sphingolipids in budding yeast *Saccharomyces cerevisiae.* The pathways and proteins responsible for the biosynthesis and degradation of sphingolipids are shown. Orm1 and Orm2 are involved in the inhibition of the activity of serine palmitoyltranferase (SPT). The transport of de novo synthesized ceramides (Cers) from the ER to the Golgi apparatus is required for complex sphingolipid biosynthesis, and vesicular transport-dependent and -independent Cer transport systems exist. The biosynthesis of myriocin, australifungin, and aureobasidin A inhibits SPT, Cer synthases, and IPC synthases, respectively. Solid arrows indicate a single metabolic step. Dotted arrow indicates that there are two or more metabolic steps. Cer, ceramide; DHS, dihydrosphingosine; LCB, long-chain base; IPC, inositol phosphorylceramide; MIPC, mannosylinositol phosphorylceramide; M(IP)_2_C, mannosyldiinositol phosphorylceramide.

**Figure 2:**
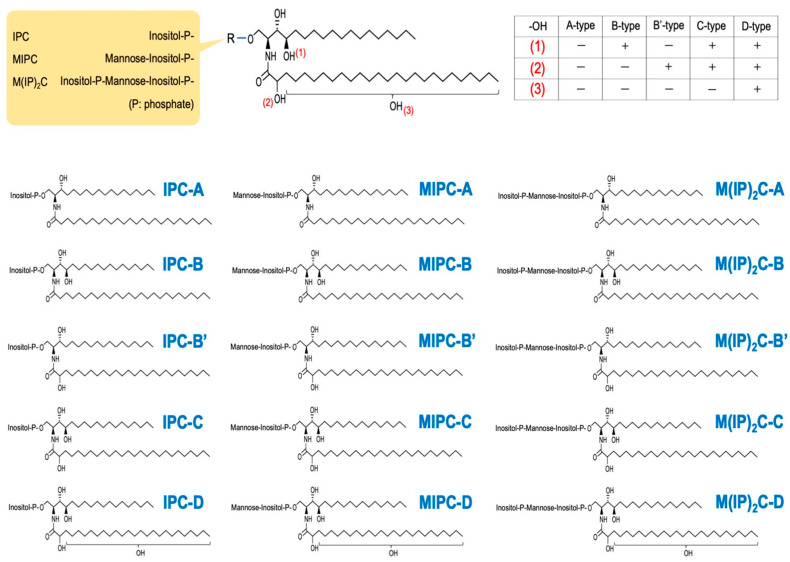
Structural diversity of complex sphingolipids in *S. cerevisiae. S. cerevisiae* has 5 types of Cer (Cer-A, B, B’, C, and D) due to the difference of hydroxylation states. Hydroxylation sites (1), (2), and (3) are the C-4 position of the LCBs, the C-2 position of the very long-chain fatty acids, and an unknown position of the very long-chain fatty acids, respectively. In *S. cerevisiae*, complex sphingolipids contain 3 types of hydrophilic group (inositol phosphate, mannosylinositol phosphate, and mannosyldiinositol phosphate), and thus, the complex sphingolipids are classified into three types (inositol phosphorylceramide (IPC), mannosylinositol phosphorylceramide (MIPC), and mannosyldiinositol phosphorylceramide (M(IP)_2_C). Therefore, 15 types of complex sphingolipids can be biosynthesized in *S. cerevisia*e through various Cer and hydrophilic head group combinations.

Similar to that in mammals, the first step in sphingolipid biosynthesis in *S. cerevisiae* is the biosynthesis of 3-ketodihydrosphingosine via a condensation reaction between serine and acyl-CoA [16,17,29]. The genes *LCB*1 and *LCB*2, which encode the serine palmitoyltransferase (SPT) that catalyzes this reaction (Figure 1), were first identified through the analysis of LCB auxotroph yeast mutant strains [12,13]. Both C18 and C20 LCBs are detected in *S. cerevisiae*, implying that yeast SPT uses both palmitoyl-CoA and stearoyl-CoA as substrates [30,31]. Yeast SPT uses serine as a substrate but can also use alanine to synthesize small amounts of 1-deoxy-LCBs [32]. Tsc3 is a regulatory subunit of SPT that plays an important role in controlling the amino acid selectivity of SPT [32]. 3-Ketodihydrosphingosine is converted to dihydroCer (Cer-A) through the action of 3-ketodihydrosphingosine reductase (Tsc10) and Cer synthases (Lag1 and Lac1) (Figure 1) [14,15]. Since Lag1 and Lac1 prefer very long-chain fatty acyl-CoA (very long chain: ≥C21) as substrates, the fatty acid chain length of Cer in *S. cerevisiae* is mostly C26 [15,28]. This structural characteristic differs significantly from that of mammalian Cers, which vary in fatty acid chain lengths centered on C12 to C24. Cers from *S. cerevisiae* are classified into five types: A, B, B’, C, and D, depending on the hydroxylation of LCBs and fatty acid moieties [28]. Cer-A comprises dihydrosphingosine (DHS) and unhydroxylated fatty acids. Sphingolipid C4-hydroxylase (Sur2) hydroxylates Cer-A at the C-4 position of the DHS moiety, forming Cer-B [33]. Cer-A and Cer-B are hydroxylated at the C-2 position of the fatty acid moiety by sphingolipid α-hydroxylase (Scs7), generating Cer-B′ and Cer-C, respectively (Figure 1) [33]. Cer-C is the most abundant type in *S. cerevisiae* [28,30]. It has been suggested that there is also a pathway in which Sur2 converts DHS to phytosphingosine (PHS), which then becomes Cer-B [33,34,35]. In Cer-D, an unknown position on the fatty acid moiety of Cer-C undergoes hydroxylation (Figure 1). Although the hydroxylase that catalyzes the conversion of Cer-C to Cer-D remains unknown, deleting *CCC*2, which encodes an intracellular Cu^2+^ transporter, abolishes Cer-D, suggesting that Cu^2+^ is required for its biosynthesis [36]. The biosynthesis of LCBs and Cers occurs in the ER, and the subsequent conversion to complex sphingolipids occurs in the Golgi apparatus [37]. Vesicular transport-dependent and -independent pathways are thought to be involved in the transport of Cers from the ER to the Golgi apparatus (Figure 1) [37]. Several factors are involved in the non-vesicular Cer transport, including Nvj2, the tricalbin proteins, and Svf1 [38,39,40]. Nvj2 is normally involved in the formation of the nuclear ER–vacuole junction; however, under ER stress or when Cer abnormally accumulates in the ER, it relocates to the ER–medial–Golgi contact site to facilitate Cer export from the ER [38]. *TCB*1, *TCB*2, and *TCB*3, which encode yeast tricalbin proteins and contribute to ER–medial–Golgi contact site formation, and the deletion of these genes reduces the non-vesicular transport of Cer [39]. However, Cer accumulation is not observed in *tcb*1∆ *tcb*2∆ *tcb*3∆ cells [39]. This is probably because Cers, which abnormally accumulate in the ER due to decreased transport from the ER to the Golgi, are converted to 1-*O*-acylCers and subsequently transferred to lipid droplets [39,41]. Svf1 contains a functional Cer-binding domain and contributes to Cer transport between the ER and the cis-Golgi [40]. Cers transported to the Golgi apparatus are converted into IPCs by Aur1 (Figure 1) [42,43]. Kei1 functions as a regulatory subunit for Aur1, and, under Kei1-repressive conditions, Aur1 is missorted to the vacuole [44]. IPCs are converted to mannosylinositol phosphorylceramides (MIPCs) by Csg1 (Sur1) and Csh1 (Figure 1) [36,45]. Csg2 has several regulatory functions for Csg1 and Csh1, including stability, transport, and gene expression, and the deletion of *CSG*2 causes a dramatic reduction in MIPC biosynthesis [45,46,47]. Finally, MIPCs are converted to mannosyldiinositol phosphorylceramides (M(IP)_2_Cs) by Ipt1 (Figure 1) [48]. Because of the presence of five Cer subtypes and three hydrophilic head types, 15 subtypes of complex sphingolipids can be biosynthesized in *S. cerevisiae* (Figure 1 and Figure 2) [28]. IPCs are abundant in the Golgi apparatus and vacuoles, but they are also distributed in the plasma membranes [49]. MIPCs and M(IP)_2_Cs are most abundant in the plasma membranes [49].

## 3. Regulation Mechanisms of Sphingolipid Metabolism

In *S. cerevisiae*, the formation of the complex between SPT and Orm1/2 and the phosphorylation of Cer synthases are well-studied regulatory mechanisms in the sphingolipid biosynthesis pathway. The regulatory role of Orm1/2 in sphingolipid biosynthesis was discovered starting with a screen for genes showing genetic interactions with *orm*2∆ or the overexpression of *ORM*1 or *ORM*2, ultimately revealing Orm1/2 as a negative regulator of sphingolipid biosynthesis (Figure 1) [16]. Under normal conditions, Orm1 or Orm2 forms a 1:1 complex with Lcb1 and Lcb2 heterodimer to inhibit SPT activity [16]. In addition, this complex contains Tsc3 and the phosphoinositide phosphatase Sac1, forming the serine palmitoyltransferase–Orm1/2–Tsc3–Sac1 (SPOTS) complex [16,50]. Orm1/2 dissociates from Lcb1/2 through phosphorylation by the yeast AGC kinase orthologs Ypk1, which results in increased SPT activity [16,51]. Furthermore, Cer also binds between Lcb1/2 and Orm1/2, collaborating with the phosphorylation of Orm1/2 to regulate SPT activity [50,52]. When a decrease in sphingolipid levels or physical stress in plasma membranes occurs, the target of rapamycin complex 2 (TORC2) is activated and subsequently phosphorylates Ypk1, resulting in the upregulation of sphingolipid biosynthesis [53]. This indicates that Ypk1-mediated Orm1/2 phosphorylation and the subsequent dissociation from Lcb1/2 serve as a feedback pathway against the impaired biosynthesis of sphingolipids. The dephosphorylation of Orm proteins via Cdc55–protein phosphatase 2A counteracts the Ypk1-mediated regulation of sphingolipid biosynthesis [54]. Furthermore, Ypk1 increases the activity of Cer synthases (Lag1 and Lac1) through phosphorylation [29], and this regulation, along with that of Orm1/2, is an important mechanism in Ypk1-mediated metabolic regulation of sphingolipids. In addition, Ypk1 suppresses the activity of Fpk1 kinase, leading to the downregulation of phospholipid flippase activity at plasma membranes [55]. Furthermore, Fpk1 also contributes to the downregulation of Ypk1. Since MIPCs are involved in maintaining Fpk1 activity, a kinase network involving specific complex sphingolipids and phospholipid asymmetry has been suggested [55]. It has been reported that Orm1/2 is also phosphorylated by Npr1, which is a kinase downstream of TORC1, and by Swe1, which is involved in cell cycle regulation [56,57], suggesting that the regulatory mechanism of sphingolipid biosynthesis via Orm1/2 is modulated by various factors. It has also been reported that the S6 kinase Sch9, which regulates ribosome biogenesis downstream of TORC1, contributes to the regulation of sphingolipid biosynthesis via Cer synthases, complex sphingolipid phospholipase C (Isc1), and ceramidases (Ypc1, Ydc1) [58]. GSK3 kinases, which are also downstream kinases of TORC1, play a role in the quantitative control of sphingolipids by phosphorylating Elo2, which is involved in synthesizing very long-chain fatty acyl-CoA [59]. In addition, casein kinase 2-mediated phosphoregulation of Cer synthases is also reported [60].

## 4. Importance of Structural Diversity of Complex Sphingolipids

### 4.1. Functional Interactions Between Specific Complex Sphingolipid Subtypes and Glycerophospholipids

The genes that have been identified so far that encode enzymes and their regulatory subunits contributing to the structural diversity of complex sphingolipids in *S. cerevisiae* include *SUR*1 (*CSG*1), *CSH*1, *CSG*2, *IPT*1, *SUR*2, and *SCS*7 (Figure 1). Single-deletion of one of these genes results in the decrease or loss of specific complex sphingolipid subtype(s) without reducing total complex sphingolipids levels, while do not show significant growth defects in a nutritionally rich media like YPD [31,61]. We found that the deletion of *SAC*1 results in synthetic lethality due to the double deletion of *CSG*1, *CSG*2, *SCS*7, or *IPT*1, suggesting functional interactions between *SAC*1 and specific subtype(s) of complex sphingolipids [62]. Phosphatidylserine (PS), which is biosynthesized in the ER, is transported to plasma membranes via the oxysterol-binding proteins Osh6/7, which requires the exchange transport of PI-4-phosphate from plasma membranes to the ER by Osh6/7 [63]. Sac1 plays an important role in maintaining the PS transport system by degrading PI-4-phosphate in the ER, and *SAC*1 deletion has been reported to reduce PS levels [64,65]. We demonstrated that the synthetic lethality caused by a double deletion in *SAC*1 and *CSG*1, *SCS*7, or *IPT*1 is caused in part by decreased PS level due to *sac*1∆. Moreover, it was also shown that there is a negative genetic interaction between *CHO*1-encoding PS synthase and *CSG*1, *CSG*2, *SCS*7, or *IPT*1 and that abnormalities in the metabolism of two different membrane lipids finally result in synthetic lethality [62,66]. In contrast, cells lacking *CSG*2 or *SCS*7 did not exhibit synthetic lethality when the biosynthesis of phosphatidylethanolamine and/or phosphatidylcholine decreased [66], suggesting that among the major phospholipids, there are particularly strong functional interactions between PS and specific complex sphingolipid subtype(s). In *CSG*2-deleted cells with moderately repressed *CHO*1 expression, the vesicular transport system from post-Golgi endosomes to the Golgi apparatus was impaired, suggesting that PS and specific complex sphingolipids cooperate to regulate specific vesicular transport [67]. On the other hand, Sac1 is reported to be involved in the modulation of sphingolipid metabolism through dephosphorylation of PI-4-phosphate to generate PI utilized for IPC biosynthesis [68]. This finding also indicates the functional interactions between complex sphingolipids and glycerophospholipids via *SAC*1.

Activation of the TORC2/Ypk1 pathway and the subsequent downregulation of Fpk1/2 decreases the activity of plasma membrane-localized phospholipid flippases, which causes activation of the Rho1-Pkc1 pathway by changing the distribution of PS in the inner leaflet of plasma membranes, thereby contributing to the stress response against reduced sphingolipids levels or physical stress in plasma membranes [69]. This result also suggests the functional connection between sphingolipids and PS.

### 4.2. Limitation of the Structural Diversity of Complex Sphingolipids by Multiple Defects in Sphingolipid-Metabolizing Genes

Defects in single metabolic steps determining the structural diversity of complex sphingolipids, which are caused by mutations such as *csg*1∆ *csh*1∆ (or *csg*2∆), *ipt*1∆, *sur*2∆, or *scs*7∆, affect sensitivity to various stresses and drugs. For example, the deletion of *CSG*2 or double deletion of *CSG*1 and *CSH*1 causes hypersensitivity to Ca^2+^, low pH conditions, or rapid cell death under nitrogen starvation [36,45,46,47,70,71,72,73]. *ipt*1∆ cells exhibit resistance to *Dahlia merckii* antimicrobial peptide 1 (DmAMP1) [74]. The deletion of *SCS*7 also affects sensitivity to certain drugs [75]. Moreover, the cytotoxic activity of syringomycin E, which is a toxin produced by the plant pathogen *Pseudomonas syringae pv. syringae*, to *S. cerevisiae* is avoided by the deletion of *CSG*1, *CSG*2, *IPT*1, *SCS*7, or *SUR*2 [76,77,78,79,80]. The deletion of *CSG*1, *IPT*1, or *SUR*2 also suppresses phenotypic defects associated with the loss of Rvs161 or Rvs167, which are N-BAR family proteins involved in regulating endocytosis and the actin cytoskeleton [81]. *sur*2∆ cells exhibit abnormality in forming lateral diffusion barriers, which confine the ER in the mother cell during ER stress [82].

**Figure 3:**
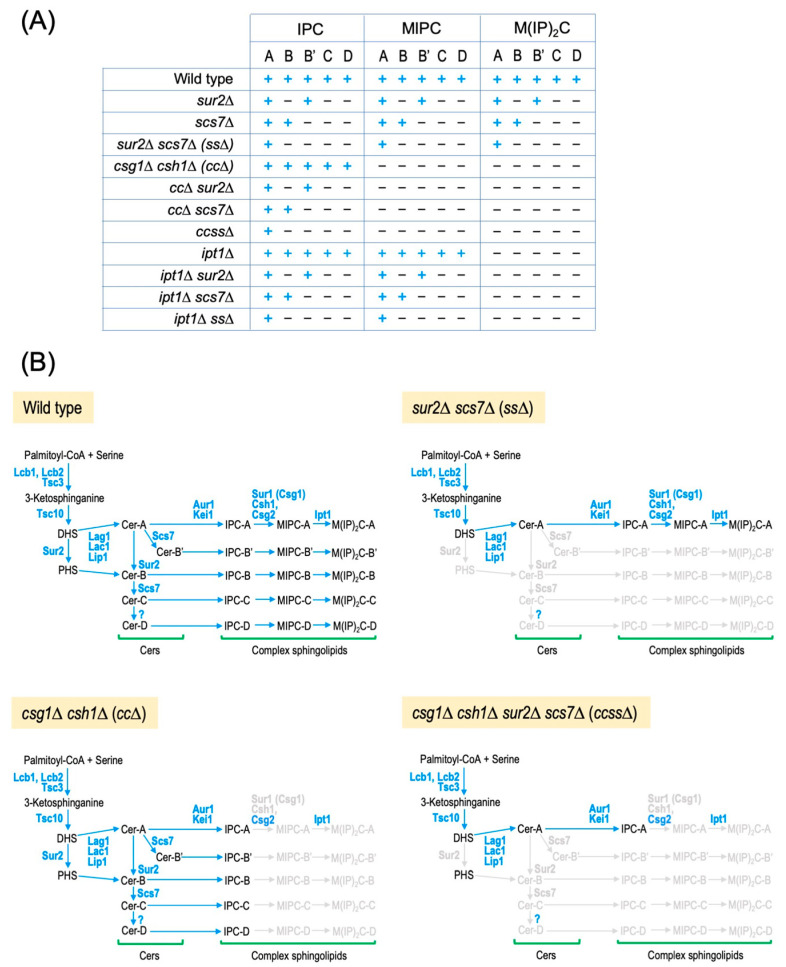
Complex sphingolipid structural diversity disruption library. (**A**) List of complex sphingolipid structural diversity disruption library. A subtype of complex sphingolipids that can be synthesized in each mutant strain is indicated by a plus symbol. (**B**) Metabolic pathways of complex sphingolipids in typical mutant strains among the complex sphingolipid structural diversity disruption library. In wild-type cells, 15 complex sphingolipid subtypes can be biosynthesized. In *sur*2∆ *scs*7∆ (*ss*∆) cells, only A-type complex sphingolipids and Cers are biosynthesized due to the loss of hydroxylation of the Cer moiety. In *csg*1∆ *csh*1∆ (*cc*∆) cells, MIPCs and M(IP)_2_Cs are not biosynthesized, and thus, IPCs are the only complex sphingolipids present. In *csg*1∆ *csh*1∆ *sur*2∆ *scs*7∆ (*ccss*∆) cells, only IPC-A is present.

Although many phenotypes of cells lacking gene-encoding enzymes (*CSG*1, *CSH*1, *SUR*2, *SCS*7, and *IPT*1) that regulate the structural diversity of complex sphingolipids have been reported, the information is somewhat fragmented. To comprehensively investigate the physiological significance of the structural diversity of complex sphingolipids in *S. cerevisiae*, we created a complex sphingolipid structural diversity disruption library, which is composed of 11 mutant cells, including combinations of deletions in *CSG*1, *CSH*1, *SUR*2, *SCS*7, and *IPT*1 (Figure 3A), and evaluated their ability to tolerate environmental stresses [31]. Among strains with losses of single metabolic steps of complex sphingolipid biosynthesis (*csg*1∆ *csh*1∆ (*cc*∆), *ipt*1∆, *sur*2∆, or *scs*7∆ cells), *cc*∆ cells that lack MIPC biosynthesis were the most impaired in the ability to tolerate stress. Two main factors likely contribute to the decreased stress tolerance of *cc*∆ cells. The first factor is suggested to be the abnormal accumulation of IPC-C due to the lack of conversion of IPCs to MIPCs. Ca^2+^ and low pH hypersensitivity and cell death under nitrogen starvation, which are observed in *cc*∆ (and also *csg*2∆) cells, can be avoided by suppressing the de novo biosynthesis of sphingolipids, which reduces IPC levels [31,47,71,72,73]. This rescue can also be achieved by the deletion of the *SCS*7 and/or *SUR*2 (*cc*∆ *scs*7∆, *cc*∆ *sur*2∆, or *cc*∆ *scs*7∆ *sur*2∆ (*ccss*∆) cells), which causes the abnormal accumulation of IPC-A, IPC-B, or IPC-B’, instead of IPC-C (Figure 3A,B) [31,47,71,72,73]. The second factor involves the loss of MIPC production itself. MIPC biosynthesis-deficient mutants exhibit impaired cell wall integrity that is not rescued by the deletion of *SCS*7 or *SUR*2 [83]. The lack of MIPC biosynthesis also causes the loss of M(IP)_2_Cs; however, the impairment of cell wall integrity is not caused by the deletion of *IPT*1 encoding M(IP)_2_C synthase [83]. Notably, the overexpression of *ERG*9, which encodes squalene synthase in the ergosterol biosynthetic pathway, rescued the impairment of cell wall integrity in *cc*∆ cells, while the repression of *ERG*9 expression exacerbated it [84]. In addition, the deletion of non-essential genes involved in the final step of ergosterol biosynthesis affected cell wall integrity in *cc*∆ cells, suggesting that MIPC and ergosterol are cooperatively involved in maintaining cell wall function [84]. In *ccss*∆ cells, in which the complex sphingolipid subtype is restricted to only IPC-A by adding deletions of *SCS*7 and *SUR*2 to *cc*∆ cells (Figure 3A,B), a tendency for increased sensitivity to various stresses, such as high osmolarity, organic acids, and various drugs, was observed as compared with *cc*∆ or *sur*2∆ *scs*7∆ (*ss*∆) cells. This suggests that the more the structural diversity of complex sphingolipids is limited, the more pleiotropic stress sensitivity tends to increase [31]. The cell wall integrity (CWI) pathway, which is a stress response pathway in budding yeast, and Msn2 and Msn4, which are general stress response transcription factors involved in various stress tolerance, are involved in the maintenance of growth of *ccss*∆ cells under normal culture conditions without stress. In addition, the CWI pathway and Msn2/4 compensated for decreased stress tolerance caused by *ccss*∆. Moreover, it was found that in *ccss*∆ cells, defects in the CWI pathway promote zymolyase sensitivity, while the deletion of *MSN*2*/*4 leads to increased plasma membrane permeability. It should be noted that *ccss*∆ cells exhibit abnormalities in lateral diffusion of plasma membrane proteins, also suggesting alteration of plasma membrane properties in *ccss***∆** cells [61]. These findings suggest that the limited structural diversity of complex sphingolipids results in hypersensitivity to pleiotropic stresses, at least partly due to abnormalities in the cell surface environment, including cell walls and plasma membranes [31].

### 4.3. Alterations in the Composition of Complex Sphingolipids in Abnormal Environments and Their Physiological Significances

*S. cerevisiae* basically prefers a slightly acidic environment (pH approximately 4–6). When the pH of the culture media deviates significantly from this environment, cell growth is seriously affected. We found that the cell growth rate and survival rate of *cc*∆ cells were significantly reduced under low pH conditions (pH 2.5–3.5) compared to the wild-type cells [71]. This phenotype was recovered by reducing the total amount of sphingolipids or the deletion of *SCS*7 or *SUR*2, suggesting that the hypersensitivity to low pH conditions is caused by the abnormal accumulation of IPC-C [71]. Is it possible, then, that *S. cerevisiae* spontaneously regulates IPC metabolism under low pH conditions? It was found that in wild-type cells, IPC levels were rapidly decreased when cells were exposed to pH 2.5 [71]. In addition, decreased expression levels of Lcb1 and Aur1 and increased expression levels of Orm2 were also observed under low pH conditions. Mutant cells overexpressing *AUR*1 and lacking *ORM*1*/*2 exhibited a significantly reduced growth rate at a pH of 2.5. These results suggest that yeast adapts to low pH conditions by spontaneously regulating the amount of IPCs [71]. Vacuolar H^+^-ATPase (V-ATPase) maintains intracellular pH homeostasis by importing H^+^ from the cytosol into vacuoles [85]. We found that V-ATPase-deficient cells were hypersensitive to the inhibition of complex sphingolipid biosynthesis [30]. Furthermore, in V-ATPase-deficient cells, dynamic changes in the composition of complex sphingolipids were observed, including a decrease in IPC levels, an increase in MIPC and M(IP)_2_C levels, and a decrease in hydroxylation of the Cer moiety. When mutations that suppress these changes were introduced into V-ATPase-deficient cells, growth at pH 7.2, at which the rate of growth of V-ATPase-deficient cells is suppressed, was further reduced. These findings suggest that when intracellular pH homeostasis is disrupted, yeast cells attempt to survive by modifying the composition of complex sphingolipids [30]. It should be noted that the elongation of the fatty acid chain length in complex sphingolipids and Cers is important for maintaining V-ATPase activity [86], which also suggests a close functional relationship between V-ATPase and sphingolipids. The deletion of *RVS*167 or *RVS*161, which are genes that encode the amphiphysin family protein, caused a decrease in the total amount of sphingolipids, which was at least partly due to increased expression of Orm2 [87]. The further suppression of sphingolipid biosynthesis in *rvs*167∆ cells alleviated the abnormal phenotypes caused by the deletion, whereas promoting sphingolipid biosynthesis had the opposite effect, implying that the proper regulation of sphingolipid levels is indispensable for amphiphysin-deficient cells [87]. Moreover, the upregulation of the biosynthesis of sphingolipids via the activation of the TORC2/Ypk1 pathway is required for the adaptive response to acetic acid stress, which also implies the importance of proper regulation of sphingolipids under environmental stress [88,89].

### 4.4. Replacing Yeast Sphingolipid Structures with Those of Other Organisms

The structure of sphingolipids can vary among different species. GlucosylCer is found in various fungi, but not in *S. cerevisiae*. However, it has been reported that the introduction of glucosylCer into *S. cerevisiae* affects the properties of the cell [90]. In the production of Japanese sake, *S. cerevisiae* and *Aspergillus oryzae* are allowed to coexist using a method called multiple parallel fermentation, in which rice starch is hydrolyzed by *A. oryzae*, and the resulting glucose is utilized by *S. cerevisiae* for alcoholic fermentation. During this process, *S. cerevisiae* acquires alkaline tolerance by incorporating the glucoyslCer generated by *A. oryzae* into the cells [90]. Interestingly, a comparative study of 90 fungal strains revealed a correlation between the presence of glucosylCer and the tolerance to alkaline conditions [91]. On the other hand, the fission yeast *Schizosaccharomyces pombe* produces IPCs and MIPCs; however, unlike *S. cerevisiae*, it does not produce M(IP)_2_Cs due to the lack of *IPT*1 [92]. When *IPT*1 from *S. cerevisiae* is expressed in *S. pombe* to produce M(IP)_2_Cs, cell death occurs, indicating that the biosynthesis of M(IP)_2_Cs is toxic for *S. pombe* [93]. These results suggest that expressing species-specific complex sphingolipids in a different species may confer stress tolerance that was not originally present or may be detrimental.

**Figure 4:**
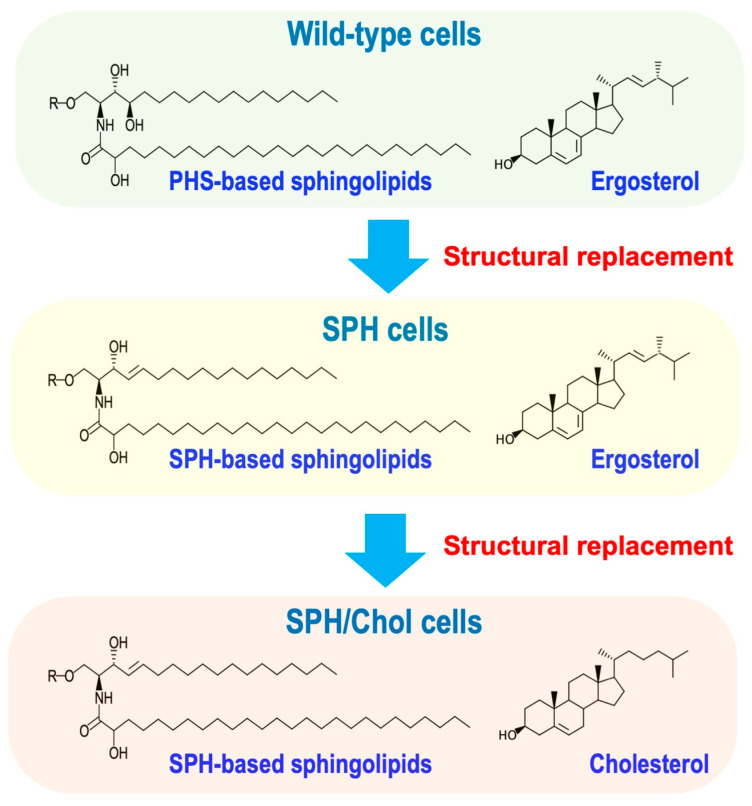
Structural replacement of LCBs and sterol in *S. cerevisiae.* The main structure of LCB in *S. cerevisiae* is phytosphingosine (PHS), while sphingosine (SPH), which is the main structure of LCB in mammals, is not biosynthesized. Furthermore, *S. cerevisiae* biosynthesizes ergosterol, whereas in mammals it is cholesterol. In SPH cells, the endogenous LCB biosynthesis pathway is completely lost, and SPH is supplied from outside the cell to synthesize Cers and complex sphingolipids, resulting in cells that have sphingolipids containing SPH [94,95]. In SPH/Chol cells, in addition to the structural substitution of LCB, the structure of sterol is also replaced by cholesterol [95].

The basic structural differences in sphingolipids between mammals and *S. cerevisiae* are even more striking. Sphingomyelin, the major complex sphingolipid in mammals, is absent in plants and fungi, including *S. cerevisiae*, which produces IPC instead. In *S. cerevisiae*, the main structure of the LCB is PHS (t18:0), whereas in mammals, it is sphingosine (SPH, d18:1), which is characterized by the presence of a trans double bond (Figure 4). Although PHS-based sphingolipids are found in some mammalian organs, *S. cerevisiae* completely lacks SPH. Furthermore, regarding sterols that are highly functionally related to sphingolipids, *S. cerevisiae* synthesizes ergosterol, while mammals utilize cholesterol. These structural differences are assumed to be the result of organisms acquiring various functions or adapting to multiple environments during evolution. However, the details remain unclear. Therefore, we investigated the abnormalities that are caused by completely changing the structure of the LCB of *S. cerevisiae’*s sphingolipids from PHS to SPH (structural replacement) [94] (Figure 4). Briefly, we constructed a system that complements the biosynthesis of Cers and complex sphingolipids by deleting the SPT gene (*lcb*2∆) in *S. cerevisiae* and supplying SPH from the medium. When SPH is supplied, SPH 1-phosphate accumulates within cells, causing cytotoxicity [23]; thus, the LCB kinase gene (*LCB*4) was also deleted (*lcb*2∆ *lcb*4∆). In cells lacking *LCB*2 and *LCB*4 and supplied with SPH (SPH cells), the biosynthesis of Cers and complex sphingolipids containing SPH was confirmed; however, SPH cells exhibited reduced tolerance to pleiotropic environmental stresses and the impaired integrity of plasma membranes and cell walls compared with the cells supplied with PHS (PHS cells) [94,95]. The phenotype observed in SPH cells may be caused by the sterol being ergosterol but not cholesterol. To further investigate this, we simultaneously replaced both the sterol and the LCB structures with mammalian types (cholesterol and SPH) in *S. cerevisiae* [95] (Figure 4). To perform the structural replacement of sterol, *ERG*1 encoding squalene epoxidase was deleted for the prevention of endogenous ergosterol biosynthesis, and a mutant form of *UPC*2 (*upc*2*-*1) that is involved in the control of the expression of sterol-related genes was expressed (*upc*2*-*1 *erg*1∆) [96]. Using a mutant strain that allows for the structural substitution of both LCB and sterol (*lcb*2∆ *lcb*4∆ *upc*2*-*1 *erg*1∆ cells), we established SPH/Chol cells with SPH and cholesterol instead of PHS and ergosterol [95]. Although the amounts of complex sphingolipids and sterols in SPH/Chol cells were comparable to those in wild-type cells, hypersensitivity to pleiotropic stresses and the impaired integrity of plasma membranes and cell walls were still observed in SPH/Chol cells, as well as in SPH cells, implying that the phenotypes observed in SPH cells are not due to structural compatibility between sterols and sphingolipids [95]. Additionally, an abnormal distribution of eisosomes, which are typical microdomains of *S. cerevisiae* [97,98], was observed in SPH/Chol cells [95]. Since the distribution pattern of eisosomes changes due to plasma membrane stress [99], the abnormality of eisosomes in SPH/Chol cells may reflect changes in the properties of plasma membranes [95]. Collectively, these results indicate that changing the LCB and sterol structures of *S. cerevisiae* to the mammalian type causes abnormalities in the cell surface environment, including plasma membranes and cell walls, ultimately decreasing stress tolerance.

## 5. Cell Death Due to Abnormal Complex Sphingolipid Metabolism and Protective Mechanisms Against It

### 5.1. Cell Growth Defect and Death Caused by Abnormal Complex Sphingolipid Metabolism

Sphingolipids are essential for yeast growth; thus, the depletion of all sphingolipids is lethal. Defects in the biosynthesis of LCBs, which are essential intermediates in sphingolipid synthesis, arise from mutations in essential genes, such as *LCB*1, *LCB*2, or *TSC*10. The deletion of *LAG*1 and *LAC*1, which encode Cer synthases, or *LIP*1, which encodes an essential regulatory subunit of Lag1 and Lac1, results in severe growth defects due to decreased biosynthesis of Cers and subsequent complex sphingolipids, although it does not result in a lethal phenotype [15,100,101]. The reason for this is that when Cer synthase activity is significantly reduced, alkaline ceramidases Ypc1 and Ydc1 can partially compensate for this defect by biosynthesizing Cers from free fatty acids and LCBs through a reverse hydrolysis reaction [20,21,100]. However, *lag*1∆ *lac*1∆ *ypc*1∆ *ydc*1∆ cells exhibit severe growth defects yet remain viable, suggesting the existence of an alternative Cer biosynthetic pathway beyond the known Cer synthase- and ceramidase-dependent pathways [100].

An abnormal accumulation of LCBs can also induce cell death. It should be noted that the repression of the biosynthesis of Cers induces the abnormal accumulation of LCBs [35,100], and australifungin, which is an antifungal agent produced by *Sporormiella australis*, inhibits yeast Cer synthase activity (Figure 1) [102]; thus, the risk of the accumulation of LCBs may exist in natural yeast populations. In *S. cerevisiae*, when LCBs are added to the culture medium, they are efficiently incorporated into cells via mechanisms that are both dependent and independent of acyl-CoA synthases Faa1 and Faa4 [103]; therefore, experiments involving the exogenous addition of LCBs are widely used to evaluate the effects of abnormal intracellular LCB accumulation. PHS added from the culture medium inhibits the uptake of tryptophan from the outside, and thus, PHS induces strong growth inhibition in tryptophan auxotrophic strains, one of the parent strains commonly used in laboratories [104,105]. The growth inhibitory activity of PHS in tryptophan auxotrophic cells is stronger than that of DHS [104]; however, in tryptophan prototrophic cells, DHS exhibits greater cytotoxicity than PHS, suggesting that DHS induces cytotoxicity through mechanisms other than the inhibition of tryptophan uptake into cells [35]. DHS-induced cell death was still observed in cells lacking genes involved in the induction of apoptosis; therefore, it was thought to be necrotic cell death. DHS also increases in mitochondrial-derived reactive oxygen species (ROS), and DHS-induced cell death is suppressed in mitochondrial DNA-deficient cells (*rho*0 cells) [35]. Furthermore, the cytotoxicity of DHS is partly mediated through the activation of the mitochondrial retrograde pathway (RTG pathway), which is involved in the upregulation of mitochondrial activity [106]. These findings suggest a close link between DHS-induced cell death and mitochondria.

An abnormal accumulation of LCB 1-phosphates, the phosphorylated form of LCBs, also causes cell death [34]. LCBs are not only converted to Cers but also converted to LCB 1-phosphates by Lcb4 and Lcb5 and are further degraded to phosphoethanolamine and fatty aldehydes by LCB 1-phosphate lyase Dpl1 (Figure 1) [8,22,23]. Then, the degradation products of LCB 1-phosphates are ultimately incorporated into the glycerophospholipid biosynthesis pathways [8]. Therefore, in double deletion mutants lacking *DPL*1 and *LCB*3 encoding LCB 1-phosphate phosphatase (*dpl*1∆ *lcb*3∆ cells), exogenously added PHS causes an excessive accumulation of PHS 1-phosphate, leading to the induction of strong growth defects, even in the tryptophan prototrophic cells [107]. Screening for suppressor mutations against exogenously added PHS-induced lethality in *dpl*1∆ *lcb*3∆ cells has shown that defects in ergosterol biosynthesis confer PHS resistance by influencing the localization and phosphorylation of Lcb4 [107].

Aur1, which converts Cers into IPCs, is also essential for cell growth [42]. The deletion of *AUR*1 results in the depletion of all complex sphingolipids and the accumulation of Cers, both of which contribute to the lethality [42]. The deletion of *ELO*3, which encodes a fatty acid elongase involved in C26 acyl-CoA biosynthesis, confers resistance to growth inhibition caused by *AUR*1 repression [108]. The effect of *elo*3∆ was not observed in growth inhibition due to the repression of Cer synthase activity, which decreases both complex sphingolipids and Cers, suggesting that it confers resistance to abnormal Cer accumulation [108]. This implies that the toxicity of accumulated Cers depends on their fatty acid chain length. Furthermore, when endogenous Cer synthases (Lag1 and Lac1) were replaced with a cotton Cer synthase that produces C18-Cer instead of C26-Cer, the deletion of *AUR*1 did not result in a lethal phenotype [109]. This indicates that IPC biosynthesis is not strictly essential for cell growth when Cer toxicity is low. However, *aur*1∆ cells expressing C18-Cer but not C26-Cer exhibit delayed proliferation and defects in cytokinesis, indicating that the production of complex sphingolipids is essential for normal cell growth [109]. In addition, the deletion of *SUR*2 or *SCS*7 also affects cell growth defects caused by the repression of *AUR*1 expression, implying that the hydroxylation states of fatty acids and LCB moieties in Cer are also important for the determination of the cytotoxic activity of Cers [110].

In mammals, Chinese hamster ovary cell mutants with thermolabile SPT (SPB-1 cells) exhibit temperature-sensitive phenotypes when sphingolipids are not exogenously supplied, indicating that as in *S. cerevisiae*, the depletion of all sphingolipids causes a growth defect on a cellular level [111,112]. Mammalian complex sphingolipids include glycolipids originating from glucosylCer or galactosylCer and sphingomyelin. The phenotypes resulting from the deletion of each biosynthetic enzyme have been reported in gene-knockout mice. Sphingomyelin synthase has two isoforms, SMS1 and SMS2, and SMS1 knockout mice exhibit moderate neonatal lethality [113]. In contrast, the deletion of SMS2 has suppressive effects on liver steatosis and obesity caused by a high-fat diet and atherosclerosis [114,115,116]. GalactosylCer synthase knockout mice exhibit abnormalities in myelin function and stability [117]. Notably, the deletion of glucosylCer synthase causes embryonic lethality in mice [118]. In contrast, ES cells completely lacking glucosylCer synthase are viable but cause abnormalities in differentiation into certain tissues [118]. Furthermore, GM-95 cells, which are a mutant of the mouse B16 melanoma cell line that completely lacks glucosylCer biosynthesis, are also viable [119]. Thus, it is suggested that glucosylCer and/or various glycosphingolipids synthesized from glucosylCer are essential for tissue formation in mice but not for the growth of single cells.

### 5.2. Mechanisms to Suppress the Inhibitory Effect of Sphingolipid Metabolic Enzyme Inhibitors That Induce Cell Death

*RSB*1, which encodes a protein belonging to the lipid-translocating export family, has been identified as a multicopy suppressor of cell growth defects in PHS-treated *dpl*1∆ cells [120,121]. Rsb1 is involved in the extracellular release of PHS and DHS, which accumulate abnormally within cells. Very recently, we have suggested that lipid-translocating export family proteins (Rsb1, Rta1, Pug1, and Ylr046c) are also involved in the extracellular release of the SPT inhibitor myriocin, which is a structural analog of LCB [122]. Through screening of multicopy suppressors against myriocin, it has been reported that Sli1 also plays a pivotal role in reducing the inhibitory effect of myriocin by acetylating it [123].

Aureobasidin A (AbA) is a cyclic depsipeptide antifungal antibiotic isolated from *Aureobasidium pullulans R*106 that inhibits Aur1 activity and exhibits strong cytotoxic activity [124,125]. The ATP-binding cassette transporters Pdr5 and Yor1 affect the acquisition of AbA resistance in *S. cerevisiae* by directly or indirectly releasing AbA accumulated within the cell to the outside of the cell [126,127]. Furthermore, we found that the overexpression of *PDR*16 and *PDR*17 attenuates the inhibitory effect of AbA on Aur1 in vivo [128]. It has been suggested that Pdr16/17 is not involved in the extracellular release of AbA and confers AbA resistance via an unknown mechanism [129]. In addition, the ability of Pdr16/17 to confer AbA resistance was exerted in ergosterol biosynthesis mutants (*erg*6∆, *erg*2∆, and *erg*5∆ cells), suggesting a functional relationship between ergosterol and Pdr16/17 [129].

### 5.3. Protection Mechanism Against Complex Sphingolipid Biosynthesis Inhibition by the High-Osmolarity Glycerol (HOG) Pathway

**Figure 5:**
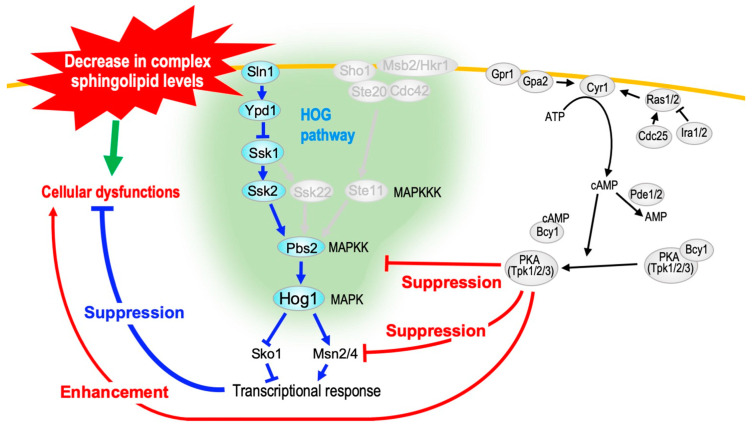
Protective role of the HOG pathway against growth defects due to the impairment of complex sphingolipid biosynthesis. When complex sphingolipid biosynthesis is repressed, the HOG pathway is activated, and the growth defect is restored by transcriptional responses mediated by Msn2/4 and Sko1 [130]. Unlike the TORC2/Ypk1 pathway, the HOG pathway compensates for cellular dysfunctions secondary to defects in complex sphingolipid biosynthesis. The HOG pathway is activated through two independent branches, the Sln1 and Sho1 branches [136], and the Sln1 branch mainly functions to rescue complex sphingolipid abnormalities. Mutations that cause the activation of PKA (*pde*2∆, *ira*2∆) promote growth inhibition under impaired complex sphingolipid metabolism via three different roots: (1) the suppression of Hog1 phosphorylation, (2) the inhibition of Msn2/4 that Hog1 does not mediate, and (3) the enhancement of the growth defect that is not mediated by Hog1 and Msn2/4 [137].

We screened suppressor mutations and multicopy suppressors that confer resistance to growth inhibition due to the repression of *AUR*1 expression, and seven genes (*SRB*8, *SSN*3, *RFX*1, *HTA*1, *XRN*1, *DCK*1, and *RAS*2) and *MSN*2 were identified as suppressor mutations and multicopy suppressor, respectively [130]. *SSN*3 and *SRB*8 encode components of the RNA polymerase II mediator complex and are involved in regulating several transcription factors, including Msn2 [131,132]. *DCK*1 encodes a guanine nucleotide exchange factor for the small GTPase Rho5, which has been suggested to be associated with hyperosmotic tolerance in *S. cerevisiae* [133,134]. Furthermore, a decrease in the total amount of complex sphingolipids activates a mitogen-activated protein (MAP) kinase pathway called the HOG pathway [135]. Moreover, Msn2 and Msn4 act as transcription factors downstream of the HOG pathway [136]. Collectively, these results suggest a close relationship between the HOG pathway and complex sphingolipids. The deletion of *HOG*1 encoding a MAP kinase of the HOG pathway promoted cell growth defects under *AUR*1-repressive conditions. This effect of *hog*1∆ was also observed under *LCB*1-repressive conditions, suggesting that defects in the HOG pathway enhance growth inhibition due to reduced complex sphingolipid levels. In contrast, the overexpression of *PBS*2 encoding MAP kinase kinase of the HOG pathway suppressed growth inhibition under *AUR*1-repressive conditions [137]. The HOG pathway did not repair the impaired complex sphingolipid biosynthesis pathway caused by *AUR*1 repression but compensates for the cellular dysfunction that occurs secondary to defects in complex sphingolipid biosynthesis (Figure 5). Thus, unlike the TORC2/Ypk1 pathway, the HOG pathway does not rescue cells by correcting the abnormal sphingolipid biosynthesis pathway. It was also confirmed that the HOG pathway does not rescue growth defects caused by abnormalities in glycerophospholipid metabolism but alleviates growth defects caused by abnormal ergosterol biosynthesis. Since sphingolipids and sterols are coordinately involved in the formation of lipid microdomains [4], it is suggested that the HOG pathway serves as a rescue mechanism against lipid microdomain disruption [130].

We also found that mutations that cause protein kinase A (PKA) activation (*ira*2∆ and *pde*2∆) promote cell growth inhibition under *AUR*1-repressive conditions, and conversely, mutations that suppress PKA activity (*ras*2∆ and *gpa*2∆) suppressed the growth inhibition [137] (Figure 5). These phenomena were partly due to the suppressive effects of PKA on Hog1 phosphorylation and Msn2/4 activation, which occur when complex sphingolipid biosynthesis is disrupted [137] (Figure 5). The HOG pathway can become cytotoxic when excessively activated. Therefore, the suppressive effects of PKA on the HOG pathway are thought to be necessary to appropriately regulate rescue by the HOG pathway, preventing it from becoming cytotoxic during disruptions in complex sphingolipid biosynthesis.

## 6. Conclusions

The loss of specific subtypes of complex sphingolipids causes the impairment of various cellular functions, indicating the physiological importance of the structural diversity of complex sphingolipids. Furthermore, under stressful conditions, yeast cells dynamically alter the composition of complex sphingolipids, which are involved in the adaptation of cells to environmental stress to protect themselves. Structural differences in complex sphingolipids across eukaryotes are important for the survival and environmental adaptation of each organism. On the other hand, transcriptional responses via the HOG pathway, CWI pathway, and Msn2/4, which are typical stress response pathways, may act as rescue mechanisms in response to quantitative and/or structural defects in complex sphingolipids. Unlike the TORC2/Ypk1 pathway, these mechanisms are not involved in repairing the impaired complex sphingolipid metabolic pathway. Therefore, yeast cells are thought to establish a robust defense mechanism against sphingolipid abnormalities by utilizing these pathways in cooperation with the repair mechanisms of sphingolipid metabolism, such as the TORC2/Ypk1 pathway. Elucidating the detailed mechanism of this interplay is important for understanding the significance of complex sphingolipids.

At present, there is no clear explanation for why the depletion of specific subtype(s) of complex sphingolipids causes pleiotropic cellular dysfunctions in *S. cerevisiae.* One possibility is that the abnormalities in the structure of complex sphingolipids affect the physical properties and dynamics of biological membranes, particularly plasma membranes. This is supported by the fact that mutant cells, which have only IPC-A as a complex sphingolipid subtype, exhibit abnormalities in permeability and lipid order in plasma membranes [31,61]. In the future, it will be necessary to approach this issue by analyzing the physical properties of plasma membranes, including the formation of lipid microdomains, in more detail. Another possibility is that specific complex sphingolipid subtypes regulate the activity of specific membrane proteins through direct physical interaction. For example, in mammalian cells, the specific interaction between the EGF receptor or insulin receptor and glycosphingolipid GM3 has been reported to regulate the activity or localization of these receptor proteins [138,139]. Although few studies on such specific interactions have been reported in *S. cerevisiae*, it is expected that using methods based on yeast genetics, such as comprehensive multiple deletions of membrane protein genes and complex sphingolipid metabolic enzyme genes, will lead to a better understanding of the physiological significance of the structural diversity of complex sphingolipids.
